# Abundance, Characterization and Diversity of Culturable Anoxygenic Phototrophic Bacteria in Manitoban Marshlands

**DOI:** 10.3390/microorganisms12051007

**Published:** 2024-05-17

**Authors:** Katia Messner, Vladimir Yurkov

**Affiliations:** Department of Microbiology, University of Manitoba, Winnipeg, MB R3T 2N2, Canada; messner1@myumanitoba.ca

**Keywords:** wetlands, marshes, aerobic anoxygenic phototrophs, purple non-sulfur bacteria, anoxygenic phototrophs, *Proteobacteria*

## Abstract

Marshes are an important ecosystem, acting as a biodiversity hotspot, a carbon sink and a bioremediation site, breaking down anthropogenic waste such as antibiotics, metals and fertilizers. Due to their participation in these metabolic activities and their capability to contribute to primary productivity, the microorganisms in such habitats have become of interest to investigate. Since *Proteobacteria* were previously found to be abundant and the waters are well aerated and organic-rich, this study on the presence of anoxygenic phototrophic bacteria, purple non-sulfur bacteria and aerobic anoxygenic phototrophs in marshes was initiated. One sample was collected at each of the seven Manitoban sites, and anoxygenic phototrophs were cultivated and enumerated. A group of 14 strains, which represented the phylogenetic diversity of the isolates, was physiologically investigated further. Aerobic anoxygenic phototrophs and purple non-sulfur bacteria were present at each location, and they belonged to the α- and β-*Proteobacteria* subphyla. Some were closely related to known heavy metal reducers (*Brevundimonas*) and xenobiotic decomposers (*Novosphingobium* and *Sphingomonas*). All were able to synthesize the photosynthetic complexes aerobically. This research highlights the diversity of and the potential contributions that anoxygenic phototrophs make to the essential functions taking place in wetlands.

## 1. Introduction

Wetlands form a significant proportion of North America’s ecosystems [1] and are defined as sites adapted to a wet environment as a result of having a water table situated near or above surface level long term as a result of poor draining of the soil [2]. This is indicated by the vegetation growth and other biological activities taking place at these locations [2]. They fulfill many systemic roles which provide stability to the surrounding locale and are an invaluable habitat for a variety of plants, fish and waterfowl [2,3]. An example of one of these roles includes their function as initial reservoirs for water during flooding, limiting damage to nearby locations [2,4]. In addition, their ecological capacity to act as a carbon sink potentially makes them a key player in combatting global warming and climate change [2]. The filtration and breakdown of anthropogenic waste and pollutants like metals, fertilizers, antibiotics and sewage has been shown to occur [2,4,5]. In fact, the many beneficial contributions have led to the development of constructed wetlands as wastewater treatment systems, important biodiversity hot zones, educational sites and for recreation [2].

In Manitoba, Canada, there are many wetlands, comprising approximately 43% of the total land area, accredited to its generally flat topography [1]. This includes swamps, bogs, fens, marshes and prairie potholes. Marshes do not form peat, have large seasonal fluctuations, are affected by ground and surface waters, usually do not have trees or shrubs and tend to be found near shallow open waters [1]. They are the minority, comprising about 2.5% of the total provincial terrestrial land [1]. Prairie potholes, also known as sloughs, are shallow ponds containing marsh-like features, abundant in Southern Manitoba [6,7].

Growing interest in understanding these ecosystems has coincided with greater research into the microbes occupying these habitats. Previously, microorganisms were found to contribute significantly to primary productivity in biofilms [8] and shown to impact carbon uptake and accumulation [9], both critical activities which researchers have primarily attributed to plants [2]. In some studies of wetland communities, eDNA sequencing of the V4 region of the 16S rRNA gene found *Proteobacteria* comprised a major group [10,11]. Analysis of their environmental contributions has mainly centered around the capability of some to participate in the biogeochemical cycling of sulfur, nitrogen and phosphorus [10,12]. However, there are also *Proteobacteria* that participate in carbon cycling and photosynthesis, undoubtedly playing a part in primary organic productivity and sequestration in marshes. This includes two groups which primarily reside in α- and β-*Proteobacteria*, purple non-sulfur bacteria (PNSB) and aerobic anoxygenic phototrophs (AAP). They utilize light energy through a cyclic pathway, where it is converted into chemical energy (ATP) through photophosphorylation with the involvement of pigment bacteriochlorophyll *a* (Bchl *a*). The process of anoxygenic photosynthesis is nearly identical in both groups, with a few exceptions: AAP only perform it aerobically and PNSB anaerobically. AAP use it as a supplemental source of energy (to cellular respiration), and PNSB have the metabolic capability to grow exclusively according to photosynthesis. Furthermore, AAP are unable to fix carbon, while PNSB can [13]. AAP and PNSB inhabit a variety of environments [13,14,15]; however, there are limited studies on their presence in wetlands [16,17,18]. Additionally, anoxygenic phototrophic bacteria’s (AnPB) contribution was not considered in previous work, as Bchl *a* measurements were not undertaken [9]. Therefore, it is important to fill these gaps of knowledge to elucidate AnPB’s impact on the critical roles that marshes fulfill, especially in terms of carbon sequestration and bioremediation.

As marsh waters are rich in organics and are well aerated due to enhanced algal and cyanobacterial oxygenic photosynthetic productivity, it was expected that AAP would be present in abundance and that the metabolically versatile PNSB may be as well. To gain insight into the composition of the culturable aerobic AnPB in wetlands, three Manitoban locations were sampled. This included a slough in King’s Park, the constructed marshes at FortWhyte Alive, both within Winnipeg, and Oak Hammock Marsh in South–Central Manitoba. These are the initial results of a long-term study on marsh water microbiology, which is the first to focus on AnPB.

## 2. Materials and Methods

### 2.1. Sample Collection and Strain Cultivation

Water was obtained on the 10 July 2023 from 8 different sites located in constructed Manitoban marshes: King’s Park, FortWhyte Alive in Winnipeg and Oak Hammock Marsh near Stonewall. The pH was taken using a broad-range pH (2.0–10.0) paper strip. Once collected, the specimens were immediately placed on ice in the dark and kept there until the return to the laboratory. Next, 10-fold serial dilutions to 10^−8^ were prepared for each sample with the following solution (g/L): MgCl_2_, 0.5; KH_2_PO_4_, 0.3; NH_4_Cl, 0.3 and CaCl_2_, 0.1, adjusted to a pH of 7.0 after autoclaving [19]. All the samples (10^0^–10^−8^) were plated onto three different media, a rich organic medium (RO), an oligotrophic medium (OM) and a potato broth medium (PM), as previously described [20]. Incubation of plates occurred at 28 °C in the dark for two weeks, which were then grown at room temperature for two additional weeks. Throughout this period, they were monitored for colored colonies of interest to streak to obtain pure cultures for subsequent analysis. Once a strain was pure, it was cryopreserved at −75 °C using OM and 30% glycerol.

### 2.2. Identification of AnPB

The colonies of interest were restreaked onto their respective isolation media. Once they were confirmed to be pure, whole-cell absorption spectra in the range of 300–1100 nm were recorded for the detection of carotenoids, Bchl *a* and light-harvesting (LH) complexes by identifying the corresponding peak(s). The plate-grown cells were resuspended in 0.3 mL of 20 mM Tris-HCl buffer (pH 7.8) and 0.7 mL of glycerol to minimize light scattering [21]. Subsequent testing (described below) of phototrophic anaerobic and photoautotrophic aerobic growth alongside 16S rRNA gene sequencing confirmed the identities of the AnPB isolates. A total of 14 strains which represented the phenotypic and phylogenetic diversity were subjected to further analysis.

### 2.3. Morphology and Physiology of Isolates

The shape and size of the cells were assessed using phase contrast microscopy (Zeiss Axioskop 2) after 4 days of growth at 28 °C in the dark on the isolation medium. Motility was evaluated in a similar manner, except after 2 days with a hanging drop slide. A Gram stain [22] as well as a KOH test [23] was checked for all the strains.

The physiological experiments were conducted at 28 °C, at a pH of 7.0, in RO for a week in the dark, aerobically and in a shaker incubator, unless stated otherwise. The temperature growth range and optimum were determined to be the following (approximate values, °C): 7, 12, 16, 20, 25, 28, 32, 37 and 41. For the pH, a range of 4.0 to 11.0 at 1.0 increments was studied. The utilization of individual carbon sources was evaluated in RO initially prepared without organics and then before inoculation, supplemented with 0.5% of the following: Na-acetate, Na-butyrate, Na-citrate, ethanol, Na-formate, fructose, glucose, Na-glutamate, lactose, malic acid, Na-pyruvate and Na-succinate. Photoheterotrophic anaerobic growth was assessed in purple non-sulfur medium (PNSM) [24]. The liquid cultures in PNSM, as well as a modified version which substituted L-cysteine and L-methionine for 1.0 mM of Na_2_S, were incubated in filled screw-capped tubes under constant light. Aerobic photoautotrophy was evaluated using liquid basal organic-free RO supplemented with 1.5 g/L of NaHCO_3_ as a carbon source and 0.5 g/L of Na_2_S_2_O_3_ as an electron donor [25] and grown under constant illumination, provided by an incandescent light bulb. To account for the organics contained within the inoculum liquid culture, 2 additional transfers of the cells into fresh basal RO were conducted to ensure the growth was truly photoautotrophic. The ability to ferment glucose, fructose and sucrose was evaluated as described [19]. Oxidase, catalase, aerobic nitrate reduction and the hydrolysis of Tween 20, 40, 60 and 80, starch, gelatin and agar were determined [19,26]. Antibiotic susceptibility was assessed with diffusion disks of the following (μg): ampicillin (10), chloramphenicol (30), erythromycin (15), imipenem (10), kanamycin (30), penicillin G (10 IU), polymyxin B (300 IU), streptomycin (10) and tetracycline (30). The strains were deemed resistant to the antibiotic if no zone of clearing was observed.

### 2.4. 16S rRNA Sequencing and Phylogenetic Study

DNA was extracted, and the partial 16S rRNA gene was Sanger-sequenced with the universal primers 515F and 806R [27]. Chromatograms were processed using DNA Baser Sequence Assembler v4.36.0 (Heracle BioSoft SRL, Mioveni, Romania) [27]. Standard nucleotide BLAST searching determined the most related species for each strain [28]. The sequences of OHM48, OHM176, OHM172, OHM24, OHM16, OHM14, KP164, FW250, KP4, FW199, FW159, FW36, FW5 and FW153 were deposited and are available in GenBank under accession numbers PP726887-PP726900, respectively.

A phylogenetic tree based on the 16S rRNA gene was constructed using MEGA v11.0.13 software [29] through neighbor joining alignment with 1000 bootstrap replicates. Its evolutionary history was inferred using the Maximum Likelihood method. The model chosen for the tree, with the Tamura-3 parameter [30], was selected using the MEGA ‘find best DNA model’ tool. A total of 62 nucleotide sequences and 1582 positions were present in the final dataset.

## 3. Results and Discussion

### 3.1. Site Description and the Isolation and Detection of AnPB

**Figure 1:**
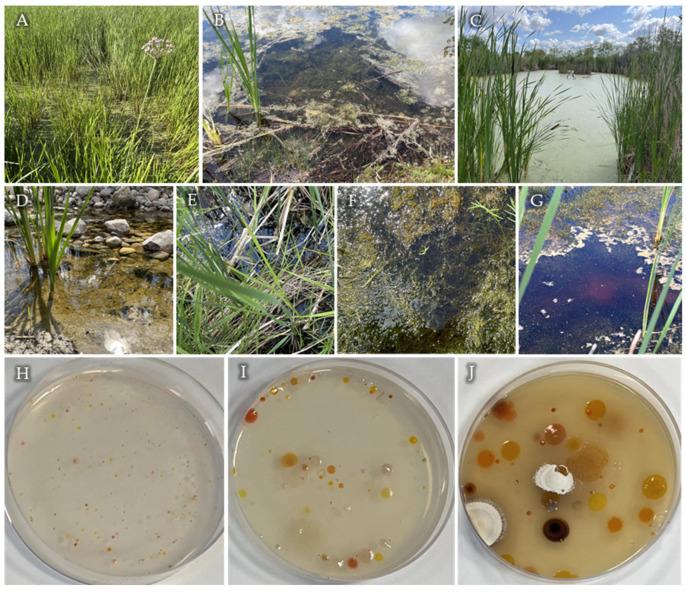
Locations sampled at Manitoba marshes. Site 1, King’s Park (**A**); Sites 2–4, FortWhyte (**B**–**D**); Sites 5–7, Oak Hammock Marsh (**E**–**G**). Colonies on OM, 10^−2^ dilution of Site 6 sample (**H**); RO 10^−4^ dilution of Site 5 sample (**I**); PM 10^−2^ dilution of Site 3 sample (**J**).

The sampling sites are shown in Figure 1. King’s Park, Site 1, is a recreational area in the South with one slough, located right beside the Red River. As marshes are typically affected by ground and surface waters [1], it is very likely that the activity along this portion of the river also impacts the slough. Site 1 was at the edge of a pond with plentiful aquatic vegetation. The water was clear, and a sample was obtained just below the surface to avoid collecting plant matter. Sites 2–4 were at FortWhyte Alive. This is a protected environment comprising forests, prairie grassland, constructed lakes and marshes, serving as a recreational and educational area [31]. In recent years, the waters have had rising phosphorus and nitrogen levels, causing eutrophication [32]. Site 2 was abundant in greenery and had floating algal–cyanobacterial mats. Site 3 was copious in macrophytes, nearly covering the entire surface. The samples were taken just below a large algal bloom and hydrophytes, if present, at each respective location. At Site 4, a thin olive-green bacterial mat from the top layer of the sediment was collected. It had rocks and more turbid water present. Sites 5–7 were at Oak Hammock Marsh in South–Central Manitoba. Formerly an extensive wetland called St. Andrew’s Bog, this 36 km^2^ area constitutes the reconstructed remnants of considerable drainage of the fertile land for agriculture [33]. Located directly beside a walkway, Site 5 had abundant reeds and floating plants at the surface. The water was slightly brown and turbid. Site 6 had an abundance of dense grass-like macrophytes. A brownish, purple sulfur bacterial mat layer on the subaqueous soil was found at Site 7. This was identified based on the smell of sulfide coming from the sample. As sulfide reacts with oxygen, it was presumed this site was anaerobic. It was agitated during collection, and as such, water directly above was acquired as well. This sample came from the deepest zone below the surface of all the sites. In general, prairie marshes are shallow [8] with anaerobic sediment at the bottom [2]; therefore, they will usually have a relatively steep oxygen gradient. As such, there is potential to find AnPB which display aerotolerant pigment production and have the capacity to conduct anoxygenic photosynthesis both aerobically and anaerobically, like the transitional *Charonomicrobium ambiphototrophicum* EG17 [25].

Surprisingly, in the three studied habitats, there was quite poor microbial mat development, suggesting the bacterial communities within the water remained mostly suspended, attached to sediments or surrounding floating plants. The sampling took place during the afternoon on a sunny day; however, the vegetation covering the surface of some sites (1, 3 and 6) may have affected the amount of light that penetrated and was available for photosynthesis. The ambient temperature (°C) was 16.5 for King’s Park, 17.6 for FortWhyte and 13.3 for Oak Hammock Marsh. The pH was taken and for each site was approximately as follows, in order from 1 to 7: 6.0–7.0, 9.0–10.0, 7.0, 7.0, 8.0, 9.0–10.0, 8.0. As these were pH paper estimations, the values reflect the range of the sites and are not exact. However, most of them fall within the expected range, as marshes are known to be relatively neutral [2]. Future experimentation will require an accurate pH meter to obtain precise values.

**Figure 2:**
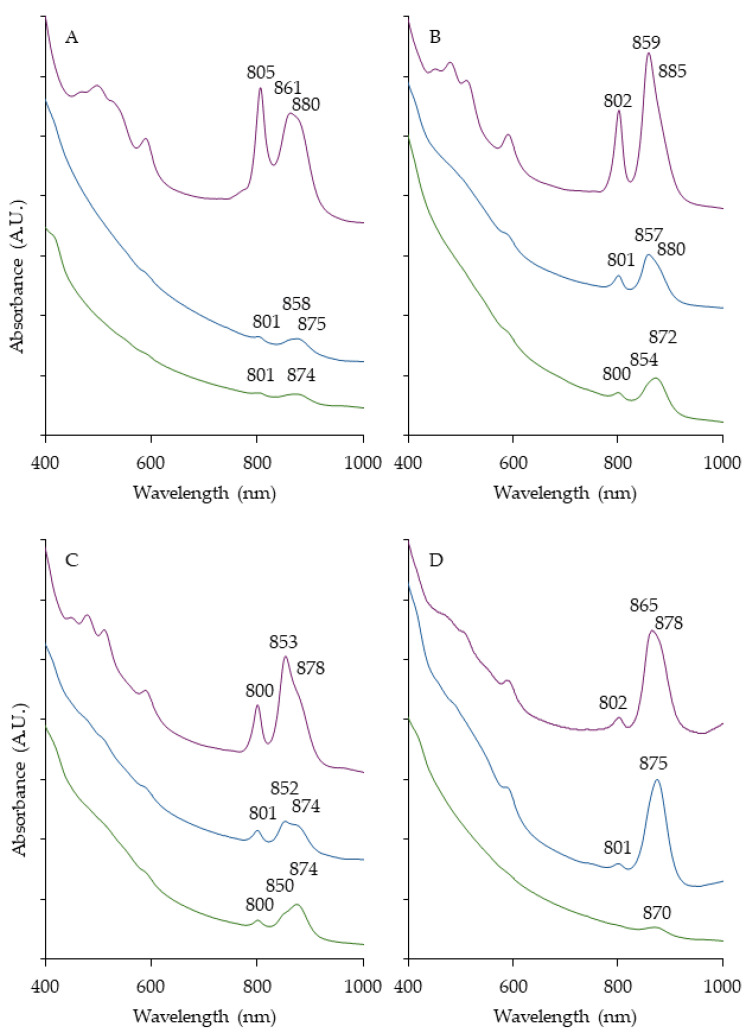
Whole-cell absorption spectra of PNSB grown at various conditions. OHM24 (**A**), KP4 (**B**), FW5 (**C**), FW36 (**D**). Spectra were taken after 4 days of growth at 28 °C in the following conditions: anaerobically in illuminated PNSM (purple) and aerobically in the dark on PM (dark blue) and RO (green). Bchl *a* peaks are indicated.

Colored colonies were present in each medium tested from all the sites, with more appearing throughout the duration of incubation. AnPB were found based on the identification of a Bchl *a* peak in the whole-cell absorption spectra [13]. In total, 102 or 43.4% of the pigmented isolates from the 235 tested were AnPB (Table 1): a total of 62.1% were in RO, followed by OM (19.6%) and PM (18.3%). The majority of the selected colonies had orange or yellow hues. From the total, 14 were selected to represent the diversity at the sites (Table 2). Four of the strains were PNSB and all were isolated on PM. This does not necessarily mean colonies did not develop on RO and OM but they were likely not isolated from these plates because PNSB usually do not actively synthesize photosynthetic pigments aerobically and produce pale colors due to limited carotenoids [14]. However, on PM, the PNSB colonies were colored and produced pigment–protein complexes (Figure 2). When these strains were plated on RO, the complexion was muted or non-colored (not shown), although they also displayed their photosynthetic apparatus (Figure 2). Therefore, based on appearance, such colonies on RO and OM were not chosen. The other 10 strains studied were AAP, confirmed according to their physiological activity. In general, this group constituted the majority of the AnPB obtained. AAP were present in all the samples regardless of depth, indicating the waters were well aerated. Support comes from the fact that these places have plentiful vegetation, algae and cyanobacteria, and as such, a significant amount of oxygen is produced from their oxygenic photosynthetic activity [2]. It is especially interesting in the case where a purple sulfur bacteria mat was observed (Site 7). It should be anoxic due to the presence of sulfide (identified by scent) as it reacts with the surrounding oxygen, therefore the majority in this community probably comprise anaerobes [14]. A possible explanation for the isolation of AAP from this site is that they were situated in the aerobic water just above the mat. In such a case, the presence of AAP nearby is proof of the presence of a steep oxygen gradient.

The presence of AnPB in the marshes aligns with previous works that identified their residence in wetland-like environments using infrared epifluorescence microscopy [16,17,34] or sampling of PNSB from soils [18]. Nonetheless, our paper is the first describing the isolation of PNSB and AAP from constructed marshes and sloughs. Obtaining pure cultures is especially important, as it allows the roles attributed to microbes to be studied directly and may help to indicate other activities the bacterial community contributes to. Here, it was used to accurately identify AnPB and distinguish between PNSB and AAP, a task difficult to perform through environmental sequencing or microscopy. Typically, sequencing of the *pufM* gene is used to indicate AAP presence in aerobic environments [35]; however, this gene is also present in PNSB, and if they are growing aerobically in the areas measured, they will also be counted. Epifluorescence microscopy [36] uses infrared lighting to distinguish AAP cells from others, but issues remain, as PNSB can also be detected using this approach. As a result, neither method precisely differentiates between the two.

### 3.2. Spectral Analysis

All the PNSB cultures (KP4, FW5, OHM24 and FW36) produced photosynthetic pigment–protein complexes anaerobically as well as aerobically (Figure 2). The Bchl *a* and carotenoid levels were higher under anoxic growth in PNSM, as expected, since light harvesting is usually conducted photoheterotrophically or photoautotrophically, where oxygen is absent [14]. Interestingly, for each isolate, the relative level of expression of LHI, LHII and carotenoids varied in the aerobic dark and anaerobic light conditions, as well as between the two media (RO and PM) in the presence of oxygen. This characteristic corresponded well with the visual difference in pigmentation in all the strains investigated, supporting the conclusion that there was varied expression of carotenoids (400–600 nm) and Bchl *a* (LH peak(s) at 850–880 nm). In general, on PM, PNSB had greater primary accessory pigment concentrations, as reported earlier [13], and greater LHII in comparison to RO (Figure 2).

Both aerobic and anaerobic photosynthetic complex expression by the same species has been seen before, although it is not common [13]. The abovementioned strain EG17, a γ-*Proteobacterium* isolated from a hypersaline spring, East German Creek, Manitoba, is to date the sole known strain which synthesizes Bchl *a* regardless of the presence of oxygen [25]. However, there are a few AAP closely related to the PNSB *Rhodobacter* [37,38], suggesting the possibility that such expression of pigments may occur in some as of yet taxonomically undefined strains. These absorption spectra do not definitively show the PNSB used light energy aerobically, but this would be worthwhile to investigate in KP4 and FW5 (related to species currently or formerly placed in the *Rhodobacter* genus [39]). FW36 interestingly showed significantly more LHI in oxic conditions than the others investigated. A close relative, *Rubrivivax gelatinosus*, has previously been shown to produce photosynthetic pigments in semi-aerobic conditions [40], and the observations in FW36 could potentially be explained by the center of colonies having less oxygen, allowing for greater expression. OHM24 synthesizing its pigments aerobically and anaerobically was the least surprising finding, considering its relation to *Rhodopseudomonas sulfidophila*, which has also displayed this characteristic [41]. As these strains, which reside in different subphyla of *Proteobacteria,* showed aerobic photosynthetic pigment–protein complex expression, it would be worthwhile to investigate whether that is the case for some other PNSB.

**Figure 3:**
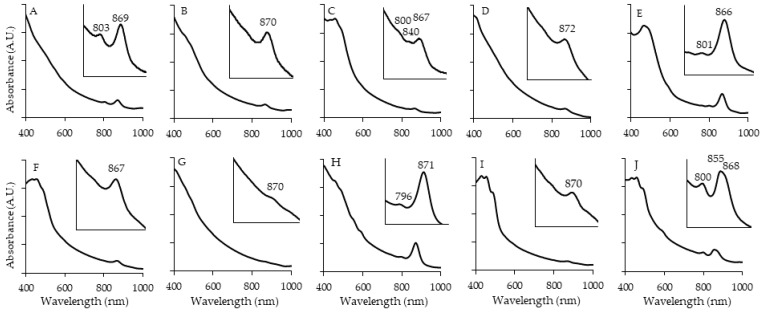
Whole-cell absorption spectra of AAP. Taken after 7 days of growth at 28 °C on their respective isolation media. Bchl *a* peaks marked. OHM14 (**A**), OHM16 (**B**), OHM48 (**C**), FW153 (**D**), FW159 (**E**), KP164 (**F**), FW172 (**G**), FW176 (**H**), FW199 (**I**), FW250 (**J**).

The AAP isolates in general displayed the typical spectral features also found in the most closely related genera (Figure 3). They all had an abundance of carotenoids relative to low Bchl *a*, which is a common attribute of the group. The majority of accessory pigments have been found to protect cells from photooxidation, and only a few support light absorbance when directly incorporated into LH complexes [13]. This was similar to the PNSB strains when grown aerobically but was vastly different to those under anoxic growth, where carotenoids and Bchl *a* were expressed in relatively equal proportions (Figure 2). FW153 displayed the most red-shifted Bchl *a* peak (872 nm) of the strains but was still within the known range [42,43]. FW159 and FW176 had the most defined LHI peaks among the isolates. FW250 and OHM48 were the only AAP with LHII produced. This has been seen previously in *Polymorphobacter* [44] and not in *Erythrobacter*, their respective closely related genera.

With the wide phylogenetic diversity of anoxygenic phototrophs isolated comprising part of the microbial community in marshes, they probably occupy an important ecological niche, although this has not yet been well investigated. Unfortunately, no studies have shown AnPB’s contribution to overall photosynthesis in wetlands, as they tend to focus on *Cyanobacteria* and chlorophyll *a* measurement [9]. Some approaches utilize the *pufM* gene, which codes for a part of the reaction center in both groups of AnPB, as a genetic indicator of AAP in aerobic environments [35]. This is an issue, as it may also capture PNSB in the oxygenated portion of the water. Therefore, it is likely such numbers are overestimated. Another strategy of AAP detection and enumeration using epifluorescence microscopy and infrared lighting [36] may also be fallible. We identified a set of PNSB occupying a wide breadth of phylogenetic diversity, including different subphyla that express their photosynthetic pigments aerobically. There is a high probability, especially in nutrient-rich locations such as marshes and high-peat-content wetlands, some PNSB will express Bchl *a* aerobically, leading them to also be detected with infrared light. Again, this would misrepresent the pervasiveness of AAP. A method that can effectively differentiate between the two with the utmost accuracy has not yet been designed, and as such, these possibilities should, at the very least, be acknowledged. Studies on the Bchl *a* prevalence and photosynthetic activity of AnPB communities would be insightful for understanding their contribution to the overall primary productivity in wetlands.

### 3.3. Phenotypic Features of the Strains

All the isolates had a gram-negative cell wall. They grew at and near a neutral pH (Table 3), as expected, since most of the sites had a pH near 7.0–8.0. No strain could survive at a pH of 5.0 or lower, and OHM14 was the only one to not grow at a pH of 6.0. The cultures from Sites 3 and 7 (FW199, OHM172, FW153, OHM176), which were alkaline (pH 9.0 to 10.0), grew at a pH of 9.0, except for OHM176. This is likely because paper tests are not very accurate, so the actual pH could be different from what was paper-estimated, as marshes are typically neutral [2], making Sites 3 and 7 unusual. The optimal growth for the group was either at a pH of 6.0 or 7.0. Interestingly, FW199, FW36 and FW5 were able to grow in significantly alkaline conditions. Aside from these instances, their growth pH range reflects the sites and what was expected from the isolates’ phylotype.

In general, the AnPB had broad temperature growth ranges, although the best for each one was at 32 or 37 °C (Table 3). FW5 and OHM16 grew at all the temperatures tested. The thermotolerance of all the representatives may be attributed to the climate of Manitoba experiencing some of the coldest and hottest temperatures in Canada annually. This is credited to its flat topography and lack of mountains, which usually act as temperature stabilizers.

As expected of the PNSB, FW5, KP4, FW36 and OHM24 grew anaerobically as photoheterotrophs. The AAP could not. All the strains were incapable of aerobic phototautotrophic growth. These two factors, in conjunction with the production of Bchl *a* (Figure 3), brought us to the conclusion that the other 10 AnPB were indeed AAP.

**Figure 4:**
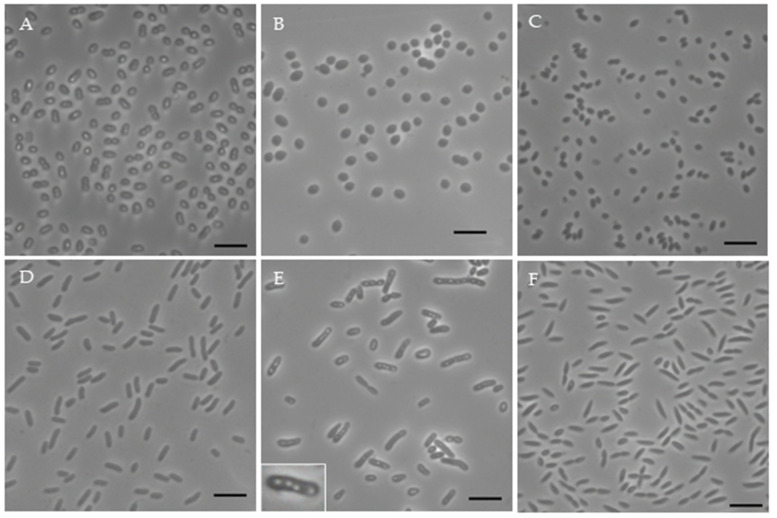
Morphology of selected strains. Included are OHM14 (**A**), FW5 (**B**), FW250 (**C**), FW36 (**D**), KP164 ((**E**), insert of cell included), FW153 (**F**). The scale bar is 5 μm.

Most of the isolates were not motile after 2 days of growth (Table 3). The strains’ morphology (Figure 4) was coccoid (FW5, OHM14), ovoid (FW250, KP4) or rod-shaped (FW199, OHM172, KP164, OHM176, FW153, OHM48, OHM16, FW159, OHM24, FW36), with FW153 having tapered ends (Figure 4F). FW5 was coccoidal, although its close relative *Cereibacter azotoformans* was characterized as ranging from ovoid to rod-shaped. KP164 had light-refractile circular globules inside it, varying from 1 to 5 per cell (Figure 4E). This could possibly be an accumulation of polyhydroxyalkanoate, which has been previously shown in some AAP depending on the conditions [45].

Most of the strains could use at least one carbon source (Table 4), except for OHM14 and FW250. Both were able to grow on RO and OM, suggesting there are essential growth components in these complex media. They were all incapable of growing with ethanol or Na-formate. Phylogenetically close groups showed similar trends. The *Erythrobacteraceae* members (OHM16, OHM48, FW159, FW172) all used Na-butyrate and Na-glutamate but not Na-citrate, fructose, lactose or malic acid. FW199, KP164 and FW153 of *Sphingomonadaceae* did not grow with Na-citrate, malic acid and Na-succinate as the sole carbon sources but could use glucose. *Paracoccaceae* KP4 and FW5 were the most versatile, utilizing Na-acetate, Na-butyrate, fructose, glucose, Na-glutamate, Na-pyruvate and Na-succinate but not lactose or malic acid. Interestingly, this also applied to FW36 but not the remaining PNSB, OHM24. FW199 was the only one able to assimilate lactose, and OHM24 was the sole strain that metabolized malic acid. Fermentation did not occur with the sugars tested. KP4 produced an acid when grown with fructose. This is typical, as only a few AnPB can ferment, and some make acids as a result of metabolizing sugars. As for enzymes, all the strains were oxidase-positive. FW199, OHN172, KP164, OHM176, OHM48 and OHM16 were catalase-positive; FW199, KP164, OHM48, OHM16, FW159 and FW36 could break down starch; KP164, FW250, OHM176, OHM16, FW159, OHM14, FW36 and KP4 hydrolyzed gelatin and FW153, FW36 and FW5 reduced nitrate into nitrite aerobically. None of them hydrolyzed agar. Strong lipolytic activity was found in the *Erythrobacteraceae* members, FW199, KP164, OHM172 and FW36. The rich variety of organic carbon types used or broken down by the AnPB (amino acids, mono-, di- and polysaccharides, organic acids, lipids) indicates their great contribution to carbon cycling and their important role in sequestering accumulated organics in marshes. Further physiological examination of pure cultures could lead to the discovery of additional influences AnPB have within wetland communities.

The AnPB had varying degrees of antibiotic resistance (Table 5); however, some general trends existed among the group. All the AnPB were susceptible to imipenem and resistant to nalidixic acid. Most were susceptible to kanamycin as well, with the exception of FW250. The PNSB isolates FW5, KP4 and FW36 had sensitivity to all the antibiotics tested, except nalidixic acid. OHM24 and FW250 resisted the highest number of antibiotics. In marshes, this is of interest, as they are recreational areas with increased human activity and therefore have greater exposure to anthropogenic waste, potentially including antibiotics. Some wetlands have also been constructed as wastewater treatment locations [2] and have been shown to break down antibiotics, primarily through the function of microbes, like some *Proteobacteria*, which use them as carbon sources [46,47,48]. Sequencing has indicated a decrease in resistance genes [46,49]. Additionally, pathogenic bacteria have been known to be dismantled through processes such as antibiotic secretion from macrophytes [50]. In contrast, it was revealed that resistance can accumulate in such environments because of selective pressure, through gene transfer and due to bacteria settling in sediments and waters, which varies by season and is dependent on the wetland type [46,48,49]. As such, the microorganisms’ performance in removing excess nitrogen and phosphorus in these habitats could be affected, as antibiotics may selectively target participants in biogeochemical cycles [46,48]. This could potentially change the community structure to more antibiotic-resistant bacteria, decreasing the total biodiversity and possibly heightening eutrophication over time [46]. Recently, concerns have been raised due to the rise in the nitrogen and phosphorus levels in the marsh waters at FortWhyte [32]. Whether this is caused by antibiotics or not, eutrophication may worsen the marshes’ capability to self-sustain, especially if important community members are outcompeted. Furthermore, when used as wastewater treatment centers, wetlands act as a point of waste release into habitats close by. If the mechanisms of inhibition are not proficient, antibiotic resistance could spread to those areas. As most of our isolates were sensitive, such persistence may also affect the relative abundance of AnPB. Even if previous studies have shown an increase in *Proteobacteria* [48], this phylum will not be universally affected the same way, as was observed in our strains. Antibiotics could limit its ability to contribute to carbon cycling and photosynthetic activity. Continual monitoring of antibiotic resistance genes in the water and among microbes within wetland wastewater treatment sites would provide greater insight into the potential impacts they may have on the habitats’ primary productivity.

### 3.4. 16S rRNA Gene-Based Phylogenetics

The results from 16S rRNA partial gene sequencing (1300–1400 bp) show most of the strains are related to AAP or PNSB (Table 2). All the isolates belong to α-*Proteobacteria*, except for a β-proteobacterium, FW36. This matches the phylogenetic placement of most known AAP and PSNB [14,51].

**Figure 5:**
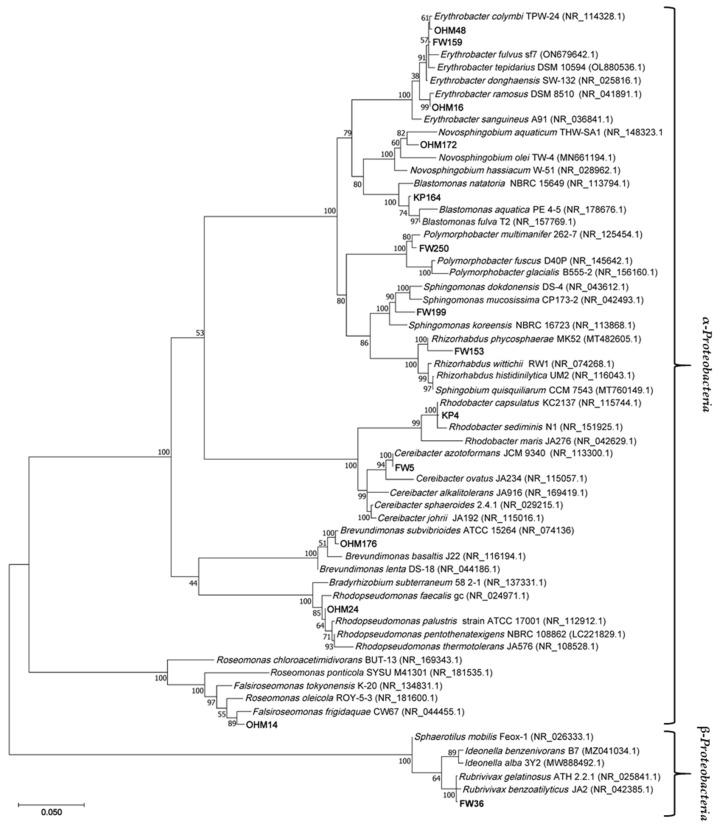
Phylogenetic tree of representative strains from Manitoba marshes and most related based on 16S rRNA gene sequences. Version with highest log likelihood (−11,688.47) is presented. Branch lengths measured as the number of substitutions per site. The percentages of trees clustered together in the associated taxa are shown next to the branches. Accession numbers for sequences used are included in parentheses.

The majority of the isolates (KP164, FW250, OHM176, OHM48, OHM16, FW159, OHM24, FW36, FW5 and KP4) likely represent new strains within most of the related species because of their very high 16S rRNA gene similarity. FW199, OHM172, FW153, and OHM14 may potentially be new species; however, they have yet to be taxonomically described as such, and DNA-DNA hybridization of the complete genome would be required to support such a conclusion, as well as finding other phenotypic distinguishing features. Four of the isolates belong to the *Erythrobacteraceae* family. OHM16, OHM48 and FW159 are all members of *Erythrobacter*, and OHM172 is from *Novosphingobium,* known AAP genera [52]. KP164, FW199 and FW153 are from the family *Sphingomonadaceae* and the genera *Blastomonas*, *Sphingomonas* and *Rhizorhabdus*, respectively. *Novosphingobium* and *Sphingomonas* representatives have been shown to degrade aromatic compounds and other xenobiotics, as well as tolerate and accumulate heavy metals [53,54,55,56]. Therefore, they may play a significant role in bioremediation and the degradation of anthropogenic waste in marshes. FW153’s most closely related genus has no AAP, and none have been shown to produce Bchl *a*. Prior reports indicated *Rhizorhabdus* did not synthesize carotenoids [57]; however, the newest member described, *Rhizorhabdus phycosphaerae*, FW153’s closest relative, proved otherwise [58]. Identification of the photosynthetic gene cluster in *Rhizorhabdus* spp. may aid in evaluating whether this genus has other AAP members or whether its features are unique to FW153. All the other strains were related to published AnPB. FW250 is the sole representative of *Polymorphobacter*, a tentative genus in *Sphingosinicellaceae* [59]. FW5 and KP4, in *Paracoccaceae*, are closely related to *Ceribacter azotoformans* (previously known as *Rhodobacter azotoformans* [39]) and *Rhodobacter capsulatus*, respectively. Both species have denitrification capabilities that were investigated for wastewater treatment [60,61]. OHM176 is associated with the *Brevundimonas* genus, found in *Caulobacteraceae*. They are resistant to high levels of heavy metals [19,62,63], possibly contributing to filtering and treating metal waste known to occur in wetlands [4]. *Nitrobacteraceae* is represented by the *Rhodopseudomonas* relative, OHM24. The most distant (based on 16S rRNA gene phylogeny, Figure 5) from all other α-*Proteobacteria* is OHM14, connected to *Roseomonas* (synonym, *Falsiroseomonas*) in *Acidobacteraceae*. FW36 was the sole strain found from the β-*Proteobacteria* subphylum. It is closely related to *Rubrivivax gelatinosus* of the *Comamonadaceae* family. Alongside contributing to photosynthetic productivity and carbon cycling, AnPB also participate in other activities, such as degrading anthropogenic pollutants and heavy metal oxides, as mentioned in the specific examples. These have been broadly advertised as important roles AAP and PNSB play in bioremediation [13,64].

## 4. Conclusions

The cultivated isolates revealed a diverse and readily available community of AnPB, indicating they are important microbial contributors to life in marsh ecosystems. This is likely because such places are highly enriched in organics, have a neutral pH and are aerobic due to oxygenic photosynthetic activity. Furthermore, the waters there are relatively shallow and have limited peat accumulation, making sunlight accessible in excess. Although these conditions better support the growth of AAP than PNSB, the metabolic flexibility of the latter has also made them possible to culture. This is especially important to consider, as many AAP studies do not factor in PNSB contributing to their relative abundance detected using infrared epifluorescence microscopy and *pufM* sequencing, leading to misjudgment of their actual abundance. However, they are indeed present, can synthesize photosynthetic pigments aerobically and therefore must be accounted for as influencing such measurements. This is one example of how cultivation is important for the precise analysis and comprehension of microbial contributions, as proof of activity can be directly assessed, and it can appropriately complement sequencing and microscopy techniques. There is a need for a better understanding of AnPB’s participation in the total primary productivity in these habitats to accurately evaluate their ecological role. Although the AnPB here represent a wide breadth of the potential community in Manitoban marshes, there are many more, which simply remain unculturable. These may include AnPB that express photosynthetic apparatus regardless of oxygen’s availability. Since there is a steep oxygen gradient in marshes, from well-aerated shallow waters to anaerobic bottom sediments, such flexibility in using anoxygenic PS would be advantageous and could exist here. Nonetheless, the phylogenetic diversity of the isolates and their known physiology provide some context to the functions they possibly perform. While some insights into the AnPB community have been enriched, more work is necessary to better elucidate their contribution to the essential activities wetlands perform for the biosphere.

## Figures and Tables

**Table 1:** Total number of isolates from Manitoba marshes.

Site	Colonies	Media			Colony Pigmentation					AnPB
		PM	RO	OM	Yellow	Orange	Red	Pink	Brown	
King’s Park										
1	38	15	18	5	8	11	10	3	1	17
FortWhyte Alive										
2	47	10	32	5	12	17	8	3	3	27
3	19	5	8	6	4	5	3	1	1	4
4	46	2	27	17	8	9	4	11	5	18
Oak Hammock Marsh										
5	31	5	20	6	6	17	3	1	1	18
6	41	4	36	1	10	16	4	4	2	14
7	13	2	5	6	1	1	1	9	0	4
Total Colonies	235	43	146	46	49	76	33	32	13	102
Overall Percentage (%)	-	18.3	62.1	19.6	20.9	32.3	14.0	13.6	5.5	43.4

**Table 2:** Representative AnPB used in this study.

Strain	Sample Site	Dilution	Media	Most Related Species	16S rRNA Similarity (%)
FW199	3	10^−3^	PM	*Sphingomonas dokdonensis* DS-4	97.69
OHM172	7	10^−2^	RO	*Novosphingobium hassiacum* W-51	98.65
KP164	1	10^−1^	RO	*Blastomonas aquatica* PE 4-5	99.47
FW250	5	10^−4^	RO	*Polymorphobacter multimanifer* 262-7	99.32
FW153	3	10^−3^	RO	*Rhizorhabdus phycosphaerae* MK52	97.11
OHM176	7	10^−2^	RO	*Brevundimonas subvibrioides* ATCC 15264	99.77
OHM48	6	10^−1^	RO	*Erythrobacter colymbi* TPW-24	99.70
OHM16	8	10^−2^	OM	*Erythrobacter ramosus* DSM 8510	99.77
FW159	5	10^−5^	RO	*Erythrobacter donghaensis* SW-132	99.77
OHM14	8	10^0^	OM	*Falsiroseomonas frigidaquae* CW67	98.87
OHM24	8	10^0^	PM	*Rhodopseudomonas pentothenatexigens* NBRC 108862	99.54
FW36	4	10^−3^	PM	*Rubrivivax gelatinosus* ATH 2.2.1	99.93
FW5	4	10^−3^	PM	*Cereibacter azotoformans* JCM 9340	100.00
KP4	1	10^0^	PM	*Rhodobacter capsulatus* KC2137	99.92

**Table 3:** Phenotypic features of representative AnPB ^1^.

Strain	FW199	OHM172	KP164	FW250	FW153	OHM176	OHM48	OHM16	FW159	OHM14	OHM24	FW36	FW5	KP4
Colony colour	Yellow	Yellow	Yellow	Yellow-Brown	Light Orange	Orange	Orange	Orange	Red	Pink	Pink	Purple-Brown	Red	Red
Cell shape	Rod	Rod	Rod	Ovoid	Rod	Rod	Rod	Rod	Rod	Coccoid	Rod	Rod	Coccoid	Ovoid-rod
Cell size (µm)	2.29 ± 0.41 × 0.63 ± 0.02	4.16 ± 0.88 × 0.72 ± 0.07	3.85 ± 0.36 × 0.81 ± 0.04	1.40 ± 0.11 × 0.78 ± 0.03	3.08 ± 0.22 × 0.68 ± 0.05	3.09 ± 0.44 × 0.60 ± 0.03	2.76 ± 0.34 × 0.65 ± 0.03	1.66 ± 0.26 × 0.87 ± 0.05	2.29 ± 0.32 × 0.58 ± 0.08	1.69 ± 0.16 × 1.18 ± 0.08	2.48 ± 0.18 × 0.82 ± 0.07	3.02 ± 0.30 × 0.72 ± 0.06	1.65 ± 0.13 × 1.31 ± 0.08	2.46 ± 0.71 × 0.84 ± 0.05
Motility	+	-	-	-	-	+	-	-	-	+	+	+	+	-
Temperature range/optimum (°C)	20–41/32	12–37/32	12–37/28	16–37/32	12–41/32	12–32/32	12–37/37	7–41/32	16–37/32	12–37/32	12–41/32	12–41/37	7–41/37	12–41/32
pH range/optimum	6–10/6	6–9/8	6–9/6	6–9/7	6–9/6	6–8/6	6–9/6	6–9/6	6–9/7	7–9/7	6–9/6	6–10/6	6–11/6	6–9/6
Enzyme Activity:														
Catalase	+	+	+	−	−	+	+	+	−	−	−	−	−	−
Oxidase	+	+	+	+	+	+	+	+	+	+	+	+	+	+
Nitrate reductase	−	−	−	−	+	−	−	−	−	−	−	+	+	−
Hydrolysis of:														
Gelatin	−	−	+	+	−	+	−	+	+	+	−	+	−	+
Starch	+	−	+	−	−	−	+	+	+	−	−	+	−	−
Tween 20	+	−	+	−	−	−	−	+	−	−	−	+	−	−
Tween 40	−	+	+	−	−	−	+	+	+	−	−	+	−	−
Tween 60	+	+	+	+	−	+	+	+	+	−	−	+	−	−
Tween 80	+	+	+	−	−	+	+	+	+	−	−	+	−	−

^1^ +, Growth; −, No Growth.

**Table 4:** Organic carbon sources utilized by representative strains ^1^.

Strain	FW199	OHM172	KP164	FW250	FW153	OHM176	OHM48	OHM16	FW159	OHM14	OHM24	FW36	FW5	KP4
Utilization of:														
Acetate	−	+	+	−	+	−	+	+	−	−	+	+	+	+
Butyrate	−	+	+	−	+	−	+	+	+	−	+	+	+	+
Citrate	−	−	−	−	−	−	−	−	−	−	−	−	+	−
Ethanol	−	−	−	−	−	−	−	−	−	−	−	−	−	−
Formate	−	−	−	−	−	−	−	−	−	−	−	−	−	−
Fructose	+	−	−	−	−	−	−	−	−	−	−	+	+	+
Glucose	+	+	+	−	+	+	+	+	−	−	−	+	+	+
Glutamate	+	+	−	−	+	+	+	+	+	−	−	+	+	+
Lactose	+	−	−	−	−	−	−	−	−	−	−	−	−	−
Malate	−	−	−	−	−	−	−	−	−	−	+	−	−	−
Pyruvate	−	+	+	−	+	+	+	+	−	−	+	+	+	+
Succinate	−	−	−	−	−	+	+	+	−	−	+	+	+	+
Fermentation:														
Fructose	−	−	−	−	−	−	−	−	−	−	−	−	−	+
Glucose	−	−	−	−	−	−	−	−	−	−	−	−	−	−
Sucrose	−	−	−	−	−	−	−	−	−	−	−	−	−	−

^1^ +, Growth; −, No Growth.

**Table 5:** Antibiotic susceptibility of AnPB ^1^.

Strain	FW199	OHM172	KP164	FW250	FW153	OHM176	OHM48	OHM16	FW159	OHM14	OHM24	FW36	FW5	KP4
Antibiotics:														
Ampicillin	R	R	S	R	R	R	R	R	R	S	R	S	S	S
Chloramphenicol	R	S	S	R	S	S	S	S	S	S	R	S	S	S
Erytromycin	S	S	S	R	R	S	S	S	S	S	R	S	S	S
Imipenem	S	S	S	S	S	S	S	S	S	S	S	S	S	S
Kanamycin	S	S	S	R	S	S	S	S	S	S	S	S	S	S
Naladixic Acid	R	R	R	R	R	R	R	R	R	R	R	R	R	R
Penicilin G	R	R	S	R	R	R	R	R	R	S	R	S	S	S
Polymixin B	S	S	S	R	S	S	S	S	S	R	R	S	S	S
Streptomycin	R	R	R	S	R	S	R	R	R	S	R	S	S	S
Tetracycline	S	S	S	R	S	S	S	S	S	S	R	S	S	S

^1^ R, resistant; S, susceptible.

## Data Availability

The data are contained within the article.
